# Intra-arterial vasodilators infusion for management of reversible cerebral vasoconstriction syndrome in a 12-year-old girl: A case report

**DOI:** 10.3389/fped.2023.1042509

**Published:** 2023-03-03

**Authors:** Frida Rizzati, Guillaume Marie, Vivianne Chanez, Thomas Ferry, Julia Natterer, David Longchamp, Guillaume Saliou, Maria-Helena Perez

**Affiliations:** ^1^ Paediatric Intensive Care Unit, Woman, Mother and Child Department, Lausanne University Hospital and Lausanne University, Lausanne, Switzerland; ^2^ Department of Radiology, Cantonal Hospital Lucerne, Lucerne, Switzerland; ^3^ Department of Radiology, Lausanne University Hospital and University of Lausanne, Lausanne, Switzerland

**Keywords:** reversible cerebral vasoconstriction syndrome, vessel wall MRI, headache, pediatric intensive care unit, children, case report

## Abstract

Reversible cerebral vasoconstriction syndrome (RCVS) is a vascular disease characterized by diffuse transient vasoconstriction and vasodilatation of the cerebral arteries. It is commonly associated with recurrent severe acute headaches with or without focal neurological deficits due to hemorrhages, infarcts, and even posterior reversible encephalopathy syndrome. The optimal management of acute neurologic deficits caused by RCVS is still uncertain. Calcium channel blockers (CCBs) such as nimodipine or verapamil have been reported to be effective in adult series. Intra-arterial injection of nimodipine, verapamil, and milrinone has recently been demonstrated to be safe and effective for treating severe segmental vasoconstriction in adults. CCBs are the most used treatment in the available pediatric literature. Intra-arterial vasodilators have been reported in some rare pediatric reports with more severe diseases, but their utility is still under investigation. We report a case of a 12-year-old girl who underwent a severe course of RCVS complicated by multiple cerebral infarcts, treated by several sessions of intra-arterial vasodilators infusion.

## Introduction

Headache is a common neurologic complaint leading to pediatric emergency room. Acute severe headache, thunderclap headache (TCH), confusion, and/or a lowered level of consciousness due to reversible cerebral vasoconstriction syndrome (RCVS) may also happen in children, even if it is excessively rare. Only a few cases are described in the literature, most of them in two main series (1, 2).

Although symptoms usually resolve spontaneously within 3 months, RCVS can be more severe and lead to permanent focal neurological deficits.

The first description of this syndrome was reported in the 1960s.

In the last few decades, RCVS has been best characterized in adult patients. Recently, Calabrese et al. defined clear diagnostic criteria for this syndrome (3). The pathophysiology of this condition is not yet completely understood and probably results from a dysregulation of cerebral vascular tone secondary to sympathetic overactivity, endothelial dysfunction, and/or oxidative stress_._ Potential triggers include exercise, cough, trauma, hypertension, and exposure to illicit drugs or serotoninergic agonists (4). Glucocorticoids worsen clinical and radiologic findings and are associated with worse outcomes in RCVS patients (5). The most severe complications of RCVS in children are cerebral infarction or intra-parenchymal hematoma, with a risk of permanent neurological deficit or even death (6).

To the best of our knowledge, this is the first pediatric case report of RCVS with a severe neurological course successfully treated with intra-arterial infusion of vasodilators.

## Case presentation

A 12-year-old girl presented to the emergency department of a secondary hospital after an episode of TCH during sports activity, associated with loss of balance and fall to the ground, followed by a short generalized clonic seizure.

Relatives also reported recurrent episodes of frontal headache of mild intensity associated with light sensitivity over the last weeks, responsive to paracetamol. She had a medical history of similar but less intense acute frontal headaches, preceded by dysarthria and/or aphasia and generalized paraesthesia, considered as migraine episodes, and successfully treated with riboflavin until 2 years ago. Family history of migraine is negative.

At the first medical assessment, she had a blood pressure of 145/98 mmHg, decreased level of consciousness with a Glasgow coma scale score of 9, and rhythmic, jerking muscle movements of the arms, suggestive of generalized clonic seizure, responsive to levetiracetam and benzodiazepine.

The electroencephalogram and native cerebral computer tomography (CT) scan were performed and were both normal. On day 2, she developed sudden onset disinhibition, left facial palsy, and left hemiplegia. The Paediatric National Institutes of Health Score (PEDNIHSS) was 3. Cerebral magnetic resonance imaging (MRI) showed several acute infarcts on the right cerebral hemisphere, with a deep middle cerebral artery (MCA) infarction in the globus pallidus and putamen. Antiplatelet therapy was introduced (aspirin 100 mg/day), and the patient was transferred to the intermediate care unit. Intravenous tissue plasminogen activator treatment was not administered because of stroke onset >4.5 h.

There was no flow on the right terminal carotid artery as observed from CT angiography and time of flight angiography (TOF) sequence on magnetic resonance angiography (MRA) on day 2. The MCA and anterior cerebral artery (ACA) were both supplied by the right posterior communicating artery (PComm). By vessel wall imaging on MRA, the pre/post-gadolinium T1-weighted SPACE sequence with fat saturation was first interpreted as a circumferential periarterial enhancement of the right supraophthalmic carotid segment with consequent suspicion of acute cerebral vasculitis. Steroid treatment (methylprednisolone 1 g/day IV) was thus introduced.

Differential diagnosis included an embolic event with occlusion of the carotid and consecutive inflammation of the wall of the vessel.

Blood workup was normal, without inflammatory markers and normal immunologic patterns. A heterogeneous pelvic mass (9.7 cm × 8.1 cm × 11.8 cm) was discovered incidentally during whole-body CT screening, which was performed because the patient had occasional abdominal pain. The patient was hypertensive, but the urinary metanephrine level and plasmatic catecholamine value were normal, excluding the hypothesis of pheochromocytoma. Control MRA was performed 1 week later, as the child presented worsening left hemiplegia (PEDNIHSS 4), revealing new ischemic areas on the diffusion tensor imaging in the right centrum semioval, visible as a fluid attenuation inversion recovery hypersignal. Stenoses and irregularities of the right MCA (M1, M2, and M3 branches) were also newly identified. Because of clinical and radiological deterioration, a switch from antiplatelet treatment to therapeutic anticoagulation by unfractionated heparin was performed on the same day, and the abdominal surgery was postponed.

On day 11, the patient developed severe left arm paresis and slurred speech with no headache (PEDNIHSS 7). She was still hypertensive with a blood pressure of 146/70 mmHg.

Transcranial Doppler confirmed indirect signs of stenosis of the right carotid, with a poststenotic flux on the right MCA, focal stenosis M1/M2 on the right MCA, reversed flow on the right ACA, and increased mean velocity on the right anterior communicating artery (AComm) and PComm and the left ACA.

**Figure 1:**
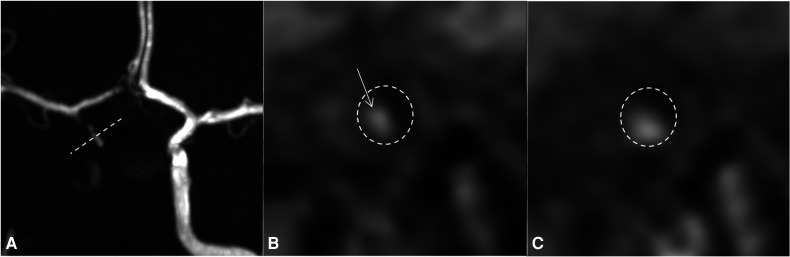
Day 4 MRI with TOF sequence (**A**) and 3D SPACE T1 imaging of the vessel wall before (**B**) and after gadolinium injection (**C**). The reconstruction of (**B**) and (**C**) images follows the dotted line on the TOF sequence (A: dotted line). For easy identification, the internal carotid artery is surrounded by a dotted circle on (**B**) and (**C**). On the TOF sequence, the right internal carotid artery is occluded and the right anterior circulation is supplied downstream by the right PComm and AComm. On vessel wall imaging, there is a spontaneous marginalized and asymmetric hyperintense signal (**B**, arrow) that enhances after injection of gadolinium, without enhancement of the vessel wall itself (**C**), highly indicative of a wall hematoma due to focal intradural dissection of the right internal carotid artery. MRI, magnetic resonance imaging; PComm, posterior communicating artery; AComm, anterior communicating artery.

All the imaging already performed was reviewed on day 11 due to the persistent worsening of patient's clinical and radiological conditions despite full anticoagulation and corticoid treatments. The review of the vessel wall imaging concludes with asymmetric vessel wall enhancement of the right supraophthalmic carotid segment evocative of a dissection of the intradural carotid artery associated with multiple focal stenoses of the cerebral arteries, suggestive of RCVS (Figure 1).

To confirm these findings, cerebral digital subtraction angiography (DSA) was performed the day after, on day 12, to achieve an intra-arterial vasodilator infusion diagnostic test. This angiography confirmed the right carotid occlusion, supplied by the AComm and PComm and multifocal subocclusive and occlusive stenoses of the right MCA and ACA.

Intra-arterial pump infusion over 30 min of 2 mg of nimodipine and 2 mg of milrinone diluted in 50 mL of physiologic serum was administered in the left internal carotid artery (ICA), which induced partial relief of the stenosis, highly suggestive of focal vasospasms. Anterograde circulation of the contrast agent was improved in the right MCA, and the two ACA and pericallosal branches occluded at the beginning of the intervention. The aspect of the occlusion of the right ICA, from the ophthalmic artery to the PComm, was more suggestive of an intracranial ICA dissection rather than a vasculopathy. The initial hypothesis of central vasculitis was thus excluded, and steroid treatment was withdrawn. The new presumed diagnosis was an intradural carotid occlusive dissection complicated by RCVS.

The patient was then admitted to the pediatric intensive care unit for continuous intravenous infusion of milrinone (0.7 μg/kg/min) and oral administration of nimodipine (60 mg six times a day).

On day 15, the MRA study showed new acute ischemia in the fronto-opercular, temporal, and pericentral right areas with slight worsening of the stenosis on the M1–M2 and A2–A3 right segments. A second intra-arterial vasodilation infusion was performed in the left ACA and the left vertebral artery (2 mg of nimodipine and 2 mg of milrinone in the two axes).

**Figure 2:**
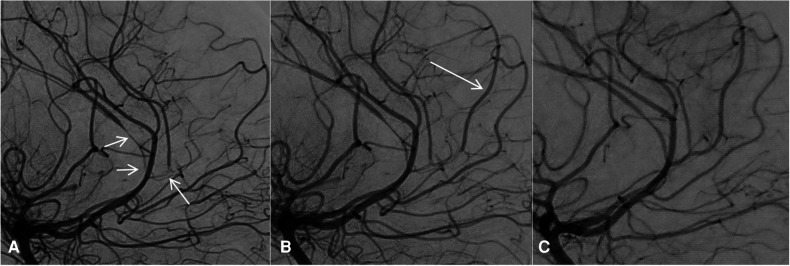
Subtracted DSA on day 12 before any vasodilator infusion (**A**) and after the first vasodilator infusion (**B**). The cerebral arteries showed diffuse and severe stenosis (**A**, arrows), which were improved after the first intra-arterial vasodilator infusion. In particular, blood flow reappeared in a pericallosal branch (**B**, arrow). On the last follow-up DSA, after the last vasodilator infusion 10 days after the first DSA, the stenosis had almost disappeared with a near-normal appearance of the cerebral arteries (**C**). DSA, digital angiography.

Due to the recurrence of neurological and radiological deterioration of the patient within 48–72 h, we decided to repeat the treatment on a routine basis every 12–24 h. In the following 10 days, the patient underwent at least daily selective intra-arterial infusions of nimodipine/milrinone under anesthesia or mild sedation, with gradual improvement of neurological status. The percutaneous arterial introducer has been kept in place to avoid several arterial punctures. Both femoral arteries were accessed: six infusions on the right side and four on the left. This kind of intra-arterial treatment was adapted from the one proposed in our radio-interventional tertiary center when vasospasm is present after subarachnoid hemorrhage due to aneurysm rupture. On day 19, the infarct burden was unchanged in MRI. The last DSA and MRI done on days 22 and 26, respectively, showed recanalization of the right terminal carotid, in line with the hypothesis of RCVS, associated with intracranial carotid occlusive dissection (Figure 2).

We performed a daily transcranial Doppler examination to monitor the degree of vasoconstriction during the intra-arterial period of treatment, confirming a progressive improvement of cerebral vasoconstriction. Milrinone continuous infusion and anticoagulation were stopped on day 39, and oral treatment of nimodipine was stopped on day 55, without any recurrence of vasospasm and/or acute stroke. On day 37, abdominal surgery was performed; the histopathology showed a mature ovarian teratoma, discarding the possible diagnosis of functional pheochromocytoma. This mature teratoma was finally not responsible for the patient's neurological situation, and it was considered an incidentaloma.

An intensive neurological rehabilitation program was initiated as soon as possible during her hospital stay with progressive functional gains. At the time of discharge from the hospital on day 114, she still presented a left hemiparesis and facial paresis, less significant than at the onset. The patient was discharged home on aspirin 100 mg daily.

## Discussion

RCVS is a rare and under-recognized syndrome, especially in the pediatric population at the acute phase (7). The misdiagnosis can lead to rapid clinical and radiological deterioration of patients, as in our case report.

The pathophysiology of RCVS remains partially unknown, but the most accepted hypothesis is a transient dysregulation of arterial tone due to sympathetic overactivity. This alteration in vascular tone may be spontaneous or evoked by various exogenous or endogenous factors. Among them, Ducros et al. mentioned extra or intracranial large artery disorders, postpartum, exposure to sympathomimetic and serotonergic drugs and tumors, endocrine factors, direct or neurosurgical trauma (6, 8), and uncontrolled hypertension (4).

The RCVS headache may be occipital or diffuse; severe and throbbing; and associated with nausea, emesis, and photosensitivity. It can recur spontaneously while the patient is at rest or can be precipitated by exertion or the Valsalva maneuver, like probably in our case during sports activity. Transient hypertension is also reported. Generalized seizures may occur during the acute period. Transient or permanent visual defects, like hemiplegia, dysarthria, aphasia, numbness, or ataxia, can occur secondary to ischemia in brain regions perfused by a severely constricted artery (3).

Uncontrolled hypertension has been mentioned in several case reports of RCVS. The etiology is still uncertain: it could be secondary to elevated intracranial pressure and affect arterial tone, triggering sympathetic overactivity and causing a vicious circle. Transient corticosteroid treatment may also have played a role in addition to the physical and mental stress of the patient.

Early discrimination between RCVS and central nervous system (CNS) vasculitis is fundamental for the choice of appropriate treatment (9).

In the present case, the first presumptive diagnosis was acute central vasculitis because of an inappropriate interpretation of vessel wall imaging on MRA on day 2, leading to the initiation of corticosteroids.

The secondary thorough review of the imaging revealed an asymmetric vessel wall enhancement of the right supraophthalmic carotid segment, more suggestive of focal intradural carotid dissection. The diagnosis of an RCVS associated with intradural dissection of the carotid artery was then confirmed later by cerebral arteriography study performance.

Radiological differential diagnosis between RCVS and central vasculitis is a dilemma in these situations. RCVS may be mistaken for primary CNS vasculitis, leading to the administration of corticosteroid treatment. The role of corticoids in the evolution of RCVS is still not clear. It has been mentioned that steroids can worsen RCVS, but this treatment (in association with immunosuppressive therapy) is the main stone of vasculitis treatment (5, 8, 10, 11). In our case, the early introduction of steroids may have played a role in the clinical and radiological deterioration of our patient.

Vessel wall imaging may reveal distinctive pitfalls, useful to characterize and discriminate pathologies involving intracranial arterial walls (9). CNS vasculitis is often characterized by multiple vessel wall thickening associated with concentric and persistent enhancement, whereas focal thickening with minimal, asymmetric, and transient enhancement is observed in RCVS. However, marked and/or persistent vessel wall enhancement is observed in a small group of RCVS, limiting the diagnostic reliability of this radiological sign (12–14).

The clinical history of a recurrent episode of TCH should tip the balance toward RCVS. Any TCH represents a medical emergency that requires early investigations, including physical and neurological examination with usually brain and cerebral vascular imaging. Associated lesions of RCVS on CT/MRI imaging are parenchymal or subarachnoid hemorrhage (0%–34%), infarction (6%–39%), and posterior reversible encephalopathy syndrome (PRES) (8%–38%) (1, 8). The first CT or MRI can be negative for typical vessel narrowing (sausage on a string) in 55–80% of situations, which was the case in our patient. The presence of infarction, potential hemorrhage, and arterial irregularity can be found in both primary CNS vasculitis and RCVS. However, circumferential enhancement of the wall of the arteries in MRI studies (T1 fat-saturated post gadolinium) is specific to primary CNS vasculitis (9, 12, 13, 15).

Management of RCVS includes treatment of the underlying cause(s) and avoidance of triggers and precipitant factors (8, 14). The patient in our case report had three potential triggers and precipitant factors for RCVS, i.e., a pelvic mass, an occluded carotid dissection, and high doses of steroids, as already discussed above. Pheochromocytoma was excluded by urinary metanephrine and plasma catecholamine levels and later by histopathology after mass resection. Retrospectively, the causative triggers of the RCVS are still of unknown origin. A potential role of the intradural carotid occlusive dissection, likely worsened by exposure to glucocorticoids, is the most likely hypothesis.

Currently, there is insufficient data to provide clear guidelines for determining the optimal treatment to achieve a lasting effect in RCVS. Each center has its empiric protocol. Nimodipine, verapamil, and milrinone have recently been demonstrated to be safe and effective when administered intra-arterially for treating severe segmental vasoconstriction in patients with RCVS. Direct intra-arterial infusion of vasodilators has an immediate effect and permits to minimize systemic hypotension (16, 17).

Although milrinone has a short duration of action, one of the advantages is the possibility of giving a prolonged IV infusion following intra-arterial use, presumably offering a more sustained effect.

To the best of our knowledge, this is the first pediatric case treated with a combination of intravenous milrinone infusion, oral nimodipine, and 10 intra-arterial infusions of vasodilators. The intra-arterial infusions were performed for both the diagnosis test and treatment of the RCVS. The choice to perform several courses of intra-arterial infusion of vasodilators, in addition to intravenous milrinone and oral calcium channel blocker (CCB) was motivated by the persistent worsening of the patient's clinical and radiological conditions under the exclusive systemic treatment. However, this should not be routinely done, and the physicians in charge should balance the risks and benefits of this treatment.

## Conclusion

We report a case of severe RCVS treated with intra-arterial infusion of vasodilators in a 12-year-old girl. The clinical and radiological distinction between cerebral vasculitis and RCVS can be difficult. Cerebral DSA and intra-arterial vasodilator infusion can be useful to differentiate these two diagnoses and consequently initiate the most appropriate treatment. The combination of intra-arterial and systemic vasodilatation seems to be an effective treatment in patients with RCVS and severe neurological deficits due to reduced perfusion.

Further research is needed to identify specific criteria for initiation of this treatment as well as effectiveness and safety in children.

## Data Availability

The original contributions presented in the study are included in the article/Supplementary Material, further inquiries can be directed to the corresponding author.
