# Supplementary figures and images for: Intra-arterial vasodilators infusion for management of reversible cerebral vasoconstriction syndrome in a 12-year-old girl: A case report
Source: Front Pediatr. 2023 Mar 3;11:1042509. doi: 10.3389/fped.2023.1042509 (PMC10020348; doi:10.3389/fped.2023.1042509)

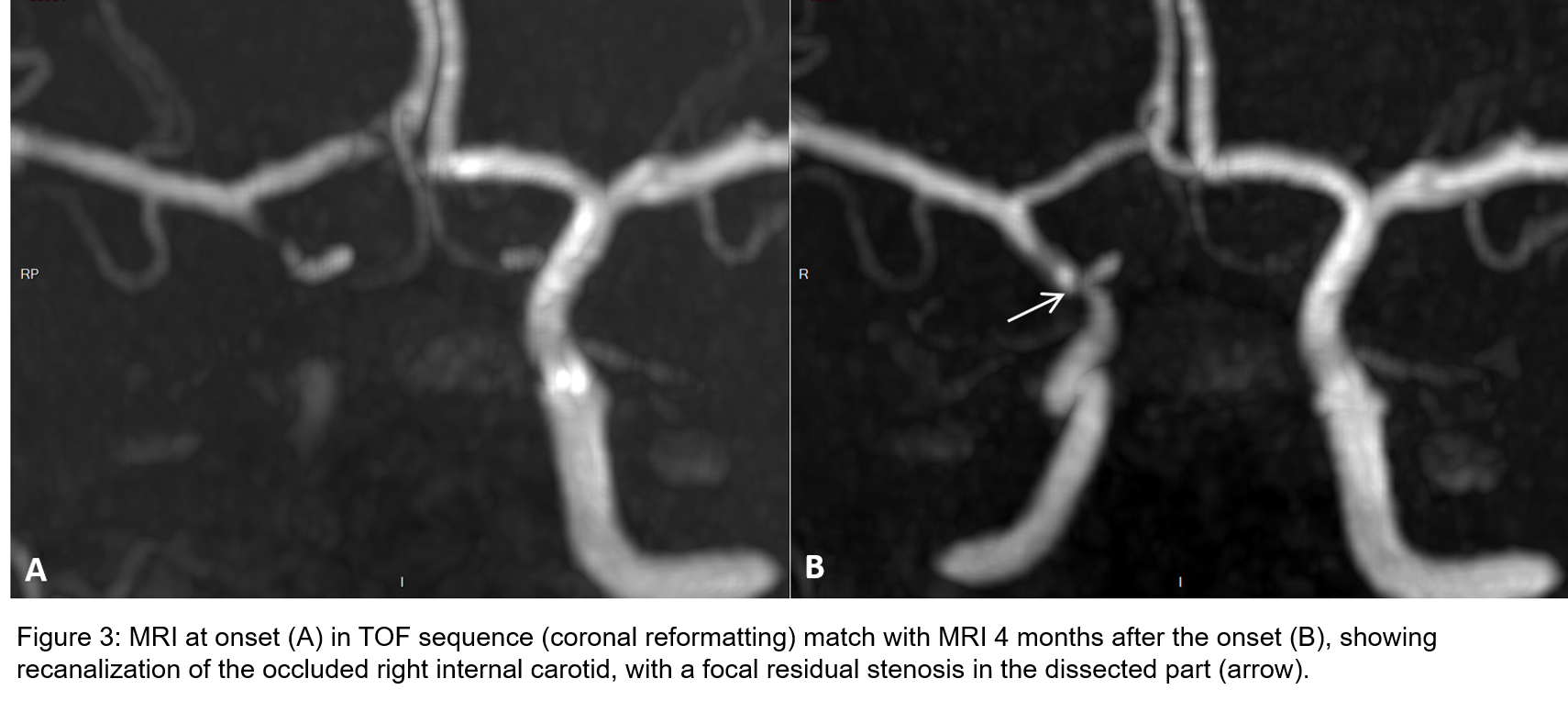

Supplement: Supplementary file 1 [file Image1.tif]
